# Semantic segmentation of COVID-19 lesions with a multiscale dilated convolutional network

**DOI:** 10.1038/s41598-022-05527-x

**Published:** 2022-02-03

**Authors:** Jianxiong Zhang, Xuefeng Ding, Dasha Hu, Yuming Jiang

**Affiliations:** College of Computer Science, Sichuan University, Chengdu, 610065 People’s Republic of China

**Keywords:** Computer science, Image processing, Machine learning

## Abstract

Automatic segmentation of infected lesions from computed tomography (CT) of COVID-19 patients is crucial for accurate diagnosis and follow-up assessment. The remaining challenges are the obvious scale difference between different types of COVID-19 lesions and the similarity between the lesions and normal tissues. This work aims to segment lesions of different scales and lesion boundaries correctly by utilizing multiscale and multilevel features. A novel multiscale dilated convolutional network (MSDC-Net) is proposed against the scale difference of lesions and the low contrast between lesions and normal tissues in CT images. In our MSDC-Net, we propose a multiscale feature capture block (MSFCB) to effectively capture multiscale features for better segmentation of lesions at different scales. Furthermore, a multilevel feature aggregate (MLFA) module is proposed to reduce the information loss in the downsampling process. Experiments on the publicly available COVID-19 CT Segmentation dataset demonstrate that the proposed MSDC-Net is superior to other existing methods in segmenting lesion boundaries and large, medium, and small lesions, and achieves the best results in Dice similarity coefficient, sensitivity and mean intersection-over-union (mIoU) scores of 82.4%, 81.1% and 78.2%, respectively. Compared with other methods, the proposed model has an average improvement of 10.6% and 11.8% on Dice and mIoU. Compared with the existing methods, our network achieves more accurate segmentation of lesions at various scales and lesion boundaries, which will facilitate further clinical analysis. In the future, we consider integrating the automatic detection and segmentation of COVID-19, and conduct research on the automatic diagnosis system of COVID-19.

## Introduction

In early 2020, coronavirus disease 2019 (COVID-19) broke out and quickly became a global epidemic, causing infections, deaths, and economic losses on a massive scale^1^. According to statistics from the World Health Organization (updated 23 February 2021), there have been 110.7 million global cumulative cases and more than 2.4 million deaths since the start of the pandemic^2^. Rapid screening of suspected patients plays a crucial role in preventing and controlling this global pandemic^3^. Reverse transcription-polymerase chain reaction (RT-PCR) is currently considered the gold standard for diagnosing COVID-19. However, with the rapid spread of the virus, RT-PCR testing faces a massive shortage of test kits and high false negative rates^4^. Computed tomography (CT) imaging can provide quantitative measurement of disease progression and has become an essential supplementary tool for RT-PCR tests to screen suspected patients and diagnose diseases^5^.

In practice, segmenting lesions form CT images can provide crucial information for doctors to diagnose and quantify lung diseases. The manual segmentation of infected regions is performed by radiologists based on their experience and suffers from inter- and intra-observer variabilities. Compared with manual segmentation, deep learning methods can automatically learn more distinguishable features from the input image, avoiding human subjectivity and other factors^6^. In recent years, deep convolutional neural networks (DCNNs) have become an important tool to assist radiologists in diagnosis^7,8^. Khan et al.^9^ explained that various CNNs have been widely used in medical image processing problems, because CNN has hierarchical feature extraction capabilities for extracting features at different levels, such as higher, mid and low-level features. For example, Chen et al.^10^ used the U-net++ network to obtain the infected regions and then classified these infected regions. Wang et al.^11^ used a more complex 3D U-Net++ network to segment the lesion regions and then used a classifier to determine whether each region is COVID-19-alike. Hassantabar et al.^12^ proposed a deep neural network and a convolutional neural network to diagnose COVID-19 patients, then a segmentation method is designed for the location of COVID-19 infected tissues in lung X-ray images. Ahmadi et al.^13^ used the Quantum Matched-Filter Technique method to find noise of MRI images and reduce it, then integrated the deep spiking neural network with conditional random field to segment brain tumors from MRI images. Khan et al.^14^ proposed a CNN-based two-stage method for classification and segmentation of COVID-19 infected areas, in which a CoV-CTNet was proposed to classify COVID-19 samples, and a segmentation model was provided to segment and analyze the infectious regions. Considering that high-quality labeled data and clean labels are usually difficult to acquire, Fan et al.^15^ proposed a segmentation network that utilizes the attention mechanism to help the network identify infected regions. Wang et al.^16^ proposed a novel framework introducing a noise-robust Dice loss function to learn from noisy labels to segment the infected regions from CT images. Zheng et al.^17^ proposed a weakly supervised deep learning framework using 3D CT volumes to detect COVID-19. Ahmadi et al.^18^ used robust principal component analysis to find brain tumor location and separate them from MRI images, then used the resulting images as ground truth images of convolutional neural network to segment brain tumors. Hussain et al.^19^ built a large dataset of chest X-ray images of COVID-19 patients, and proposed a CNN-based method to discriminate COVID-19 patients from healthy individuals. Moreover, in the work proposed in^20^, the authors use machine learning algorithms to evaluate the effect of statins on the severity of COVID-19 based on clinical characteristics, and concluded that decision tree is an effective method for predicting the severity of COVID-19.

**Figure 1:**
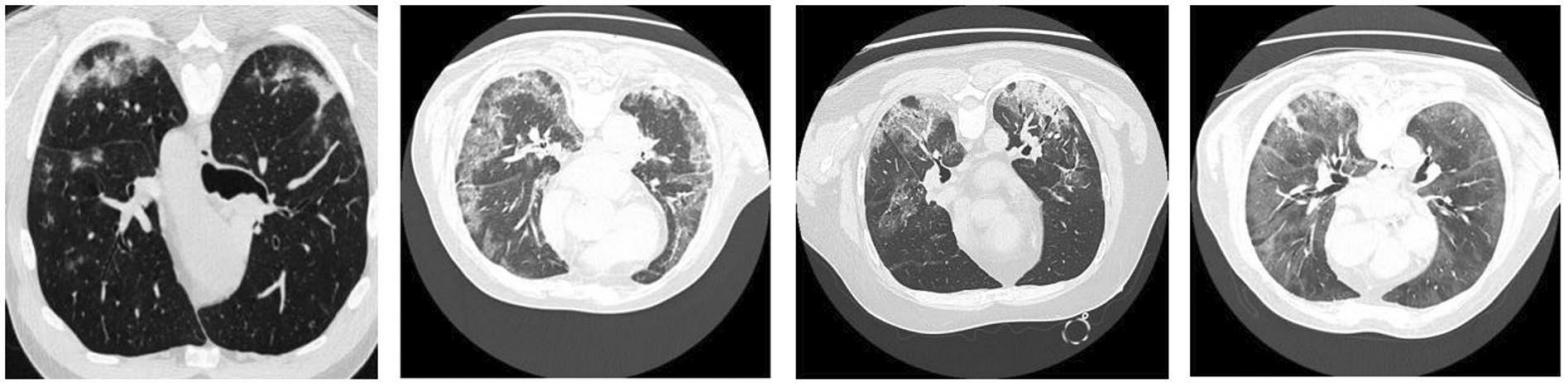
Samples and corresponding labels, where the ground-glass, consolidation, and pleural effusion are marked in dark gray, bright gray, and white, respectively.

Although some methods have been proposed to segment infected lesions from CT images, the difficult problems have not been completely solved. The scale of different infected lesions varies greatly in CT images. As shown in column 4 of Fig. 1, the ground glass occupies almost the entire lung area, while the size of the pleural effusion is only more than 10 pixels. Our motivation stems from the fact that existing methods ignore the importance of multi-scale features for segmentation of objects of different sizes. Therefore, the network needs to acquire the image features of lesions at different scales^21,22^, which have a great influence on the segmentation accuracy^23^. These multiscale features will determine the accuracy of pixel classification during the lesion segmentation. In fact, the above methods do not fully consider the multiscale feature information of infected lesions. Moreover, it can also be seen from Fig. 1 that the appearance of infected lesions is quite similar to that of normal tissues on the same CT. To accurately segment the lesion boundaries, the above methods usually use skip connections to recover the detailed information during the upsampling process, while ignoring the downsampling process. To address above issues, we proposed several key modules in our multiscale dilated convolutional network (MSDC-Net) to gather and integrate more multiscale information and replenishing the loss of context information in downsampling operations.

**Figure 2:**
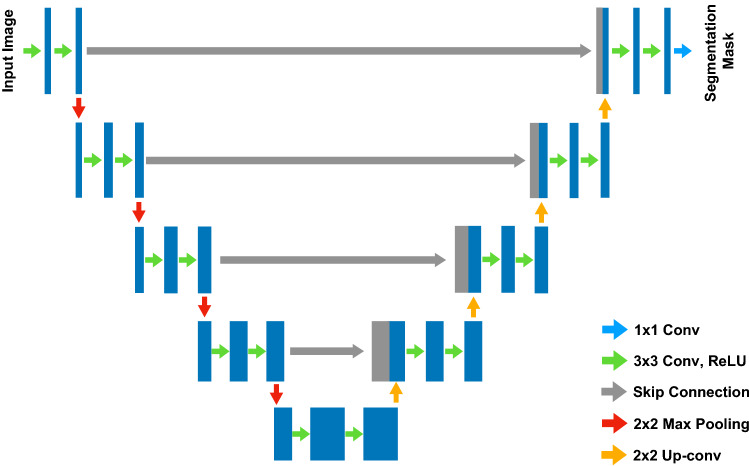
The architecture of U-Net. The network consists of a downsampling path and an upsampling path.

In this paper, we propose a MSDC-Net for precise segmentation of infected lesions from COVID-19 chest CT. Our motivation stems from the fact that the multiscale features contribute the network to accurately segment objects of different sizes and detailed information in low-level features promotes network to segment object boundaries accurately. Inspired by the excellent performance of^24–26^, shown in Fig. 2, we also use an encoder–decoder structure but extend it with several key components. In contrast to the above methods, we take advantage of the multiscale and multilevel features to improve the segmentation of infected regions at different scales and lesion boundaries. The dilated convolution is introduced in the downsampling path to extract more extensive context information. Meanwhile, a multiscale feature fusion (MSFF) module is proposed to fuse features captured by previous layers in a more effective way, which allows the network to capture multiscale features of lesions. Furthermore, unlike existing methods usually only utilize low-level features in the upsampling path, we propose a multilevel feature aggregate (MLFA) module to aggregate the features of different levels before upsampling to reduce the loss of spatial and structural information.

In this work, we focus on multiscale and multilevel feature learning. To sum up, the contributions of this paper are as threefold:

1. We propose a multiscale feature capture block (MSFCB) which employs a series of dilated convolutions to capture contextual features at different scales and a MSFF module to fuse the learned features of different convolutional layers in a more effective way.
2. A MLFA module is used to aggregate feature learned in several different blocks. Therefore, a reinforced aggregation of low- and high-level features is used to improve the accuracy of segmentation, especially for lesion boundaries.
3. We propose a novel MSDC-Net for precise segmentation of infected lesions at different scales and lesion boundaries with a combination of MSFCB and MLFA module. Extensive experiments on COVID-19 CT segmentation dataset demonstrate the effectiveness of the proposed MSDC-Net.

The rest of the paper is organized as follows. The framework of the proposed MSDC-Net and the structure of various modules are presented in section “Methods”. In section “Experiments and analysis”, the performance of the proposed module is evaluated and verified and the performance of the proposed model is compared with other state-of-the-art models. We then discuss the experimental results and the limitations of this paper in sections “Discussion” and “Limitations”. Section “Conclusion” concludes the paper.

## Methods

In this section, we first introduce the dilated convolution in details. We then present the architecture of our MSDC-Net and MSFCB and clarify how to use them to extract multiscale features. Finally, we provide the details of our MSFF module and MLFA module and illustrate the working process.

**Figure 3:**
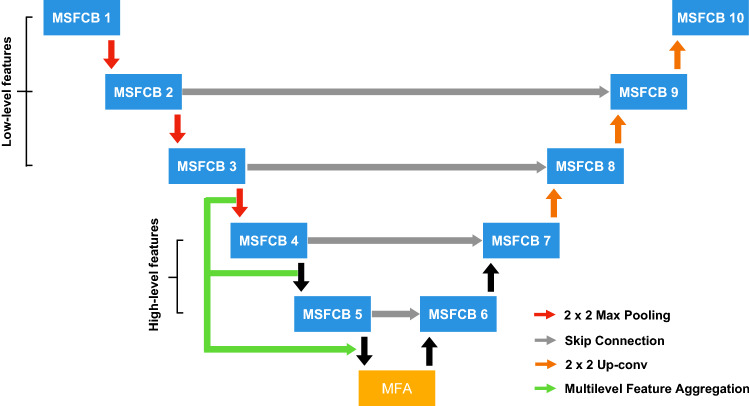
Proposed MSDC-Net architecture. The convolutional blocks are replaced by the proposed MSFCB. Moreover, an MLFA module is proposed to aggregate features of different levels in the downsampling process, instead of using only low-level features during upsampling.

Our proposed MSDC-Net is shown in Fig. 3. First, differently from^24,27,28^ that only use regular convolution for extracting features, we introduce a new MSFCB, which uses dilated convolution to gather context information of different scales. Then, we add an MSFF module at the bottleneck of the MSFCB, where the MSFF module introduce parallel inter-linking among dilated convolutions to fuse multiscale features. Finally, to better segment boundaries of lesions, we add a MLFA module at the bottleneck of the encoder–decoder structure to aggregate low- and high-level features though using inter-linking among different MSFCBs.

### Dilated convolution for multiscale feature extraction

**Figure 4:**
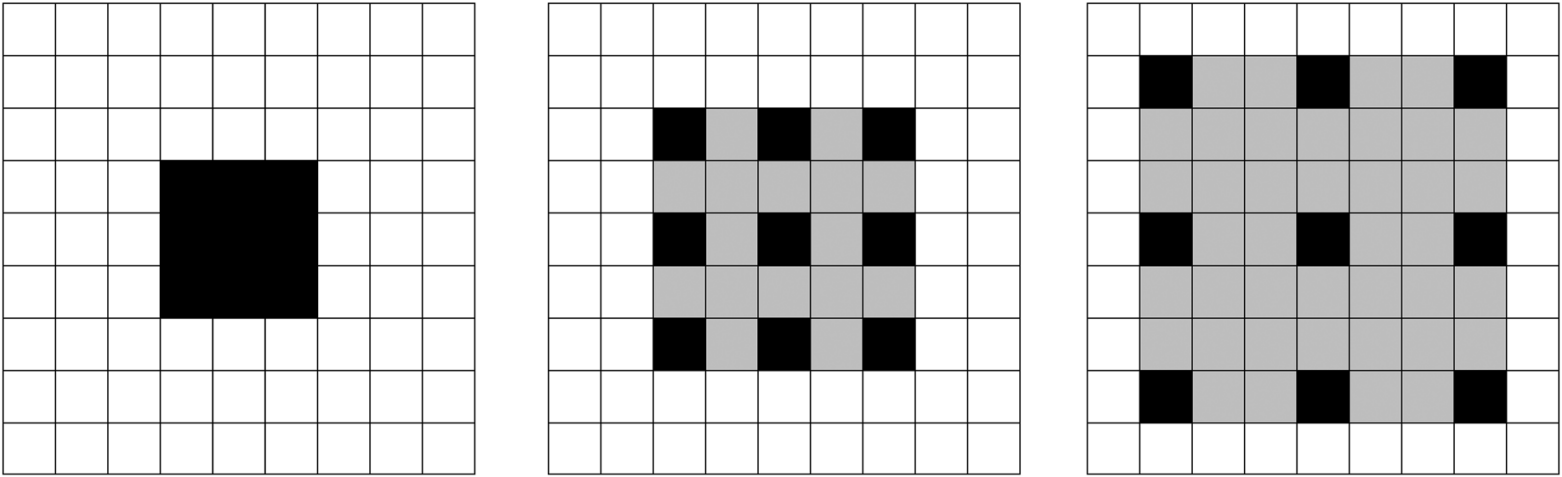
Comparison of receptive fields between regular convolution and dilated convolution. From left to right: (1) regular convolution, (2) dilated convolution (*r* = 2), and (3) dilated convolution (*r* = 3).

As shown in Fig. 4, the black squares represent the elements of the kernel. Compared with a regular convolution with size of 3 × 3, the dilated convolution with rate *r* enlarges the kernel size to $(2r + 1) \times (2r + 1)$ by inserting holes in the filter. This allows the network to capture extensive context information of the COVID-19 lesions.

### MSDC-Net model and MSFCB architecture

The architecture of our MSDC-Net is shown in Fig. 3. We use the proposed MSFCB to replace the sequence of two regular convolutions in the original U-Net. The first three blocks are composed of regular convolutions, and the last two blocks are composed of dilated convolutions with different rates. In addition, we use the MSFF module in each block to effectively fuse the features learned from different layers to obtain multiscale features. As shown in Fig. 3, the input images are fed to first three blocks to extract low-level features with high-resolution detail information. Then, the extracted features are input into last two blocks, where a series of dilated convolutions with different rates are utilized to obtain the complement of the receptive field, capturing high-level features with context information. Then, we use a MLFA module to aggregate features learned in several different blocks to replenish information loss in the final feature map. Considering that the dilated convolution can enlarge the receiving field without increasing the parameters, we remove the pooling layer after the last two blocks to further reduce the loss of information in the downsampling process.

**Figure 5:**
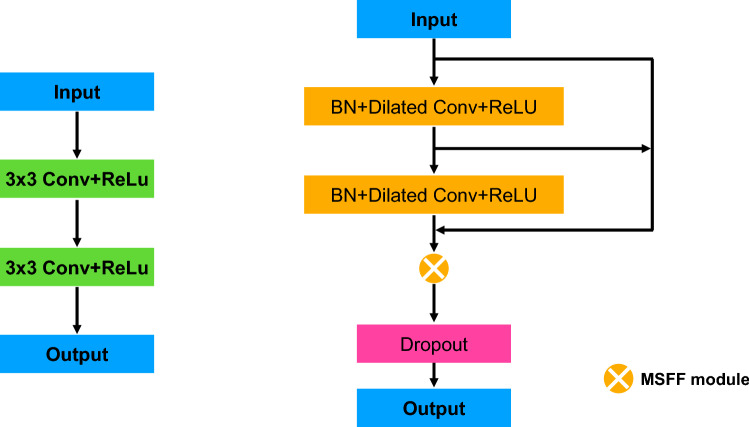
The architecture of multiscale feature capture block (MSFCB).

In contrast to previous studies^8–11^ which simply use two 3 × 3 regular convolutions, we use a novel MSFCB to effectively capture multiscale features of lesions, as shown in Fig. 5. Therefore, it allows the network to improve the accuracy of network segmentation, especially for lesions of different scales. We also add batch normalization before the convolution operation to speed up the convergence of the network by using much higher learning rates. The input of MSFCB is a feature map generated by previous MSFCB or input image. As shown in Fig. 5, dilated convolutions with different receptive fields are used to cover the corresponding size features, which can capture lesion features of various scales. Then, we use MSFF module to obtain multiscale lesion features by fusing the features learned at different scales. At the end, we add a Dropout to randomly drop units from the neural network during the training. This can avoid overfitting when network with a large number of parameters or a small amount of training data.

### Multiscale feature fusion (MSFF) module

Compared with the existing methods, we add a MSFF module to fuse the output of all the layers in the block after the last layer. Let *x*_*l*_ be the output of the last layer in the block:

$$x_{l} = H(x_{l - 1} ) \otimes H(x_{l - 2} ) \otimes H(x_{l - 3} )$$

where *H* is defined as a batch normalization (BN), followed by a 3 × 3 convolution and a ReLU, and $\otimes$ represents the feature fuse operation.

**Figure 6:**
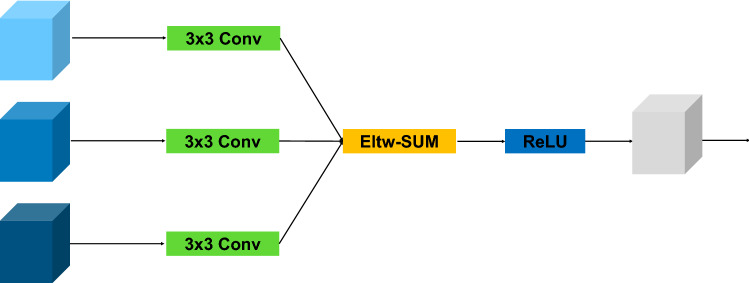
Illustration of the multiscale feature fusion (MSFF) module. Eltw-SUM refers to point-to-point addition of feature maps containing different levels of features.

Different from the simple fusion method of existing methods, we propose a more effective fusion method. As shown in Fig. 6, we use three 3 × 3 convolution layers to learn from the feature maps of different dilated convolutions before fusion for a better fusion effect. Then, three feature maps generated by the different dilated convolutions are summarized point to point. Finally, we used a ReLU to reduce the interdependence between parameters and alleviate the occurrence of overfitting problems. This module fuses features captured by dilated convolutions to obtain the multiscale features but also permits the gradient to flow directly to earlier layers, which makes the network easy to train.

### Multilevel feature aggregate (MLFA) module

Several works have shown that detailed information in low-level features helps network to segment object boundaries. However, most existing methods usually use all low- and high-level features in the upsampling path. Hence, we aggregate the features of different levels in the downsampling path with a MLFA module, as shown in Fig. 3. Features learned in different layers are aggregated to alleviate the loss of information in the downsampling process, so that the final feature map contains more spatial and location information.

**Figure 7:**
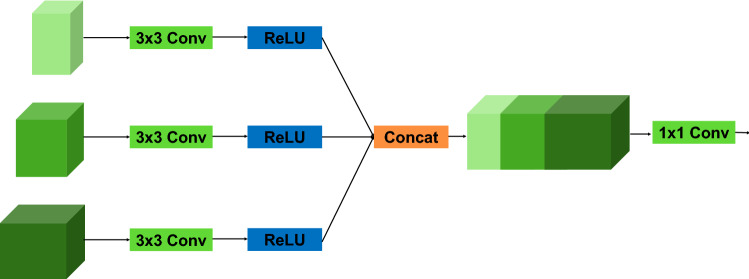
Illustration of the multilevel feature aggregation (MLFA) module.

As shown in Fig. 7, three feature maps containing different levels of features are concatenated along their channel axis. The semantic information contained in these feature maps is quite different, and the features of each level are critical to the segmentation of the boundaries. So, we concatenate them along their channel axis to obtain more semantic information. Then, three 3 × 3 convolutions are used to learn features from feature maps adaptively for better fusion effects. After concatenating the feature maps in the above manner, we employ another 1 × 1 convolution to reduce the channels of the fusion results and recombine features, avoiding the possible heavy computation complexity and memory footprint.

## Experiments and analysis

### Dataset description

In this work, we use the publically available “COVID-19 CT Segmentation dataset”^29^, which contains 100 axial CT images from 40 different COVID-19 patients. The resolution of images is 512 × 512. Segmentation was performed by a radiologist using three labels: dark gray, bright gray, and white. To avoid overfitting, we augment the original dataset using flips and rotations, and the resolution of images is still 512 × 512. We randomly divide the dataset into three sub-sets: training set, test set and validation set, the proportions of which are 50%, 30%, and 20% respectively.

### Experimental setup

#### Loss function

We adopted the categorical cross entropy loss function:

$$J(\theta ) = - \frac{1}{M}\sum\limits_{x = 0}^{M} {\sum\limits_{i = 0}^{N} {\sum\limits_{j = 0}^{C} {y_{ij}^{(x)} \log p_{ij}^{(x)} } } }$$

where $\theta$ is the set of parameters of the model, *M* denotes the number of samples, *N* denotes the number of pixels, *C* denotes the total number of categories, and *y* corresponds to the one-hot encoding of the sample label. *p*_*ij*_ is calculated by Softmax, which denotes the probability of assigning the label *i* to the pixel *j*. The network is trained by using adaptive moment estimation (Adam) to minimize the loss function. Compared with stochastic gradient descent, the Adam optimization algorithm designs independent adaptive learning rates for different parameters by calculating the first-order and second-order moments of the gradient.

#### Evaluation metrics

For a quantitative evaluation, we use the four widely adopted metrics, i.e., the mean intersection-over-union (mIoU), Dice similarity coefficient, sensitivity, and specificity.

$$\begin{gathered} mIoU = \frac{1}{k + 1}\sum\limits_{i = 0}^{k} {\frac{pii}{{\sum\nolimits_{j = 0}^{k} {pij + \sum\nolimits_{j = 0}^{k} {pji - pii} } }}} \hfill \\ Dice\;Score = \frac{2TP}{{2TP + FP + FN}} \hfill \\ Sensitivity = \frac{TP}{{TP + FN}} \hfill \\ Specificity = \frac{TN}{{TN + FP}} \hfill \\ \end{gathered}$$

where *k* represents the number of pixel categories, *p*_*ii*_ represents the number of pixels whose actual category is *i* and the predicted category is also *i*, *p*_*ij*_ represents the number of pixels whose actual category is *i* but predicted category is *j*, and *p*_*ji*_ represents the number of pixels whose actual category is *j* but predicted category is *i*. Furthermore, *TP*, *FP*, and *FN* represent true positive, false positive, and false negative predictions, respectively.

### Ablation study

In this subsection, we conduct several experiments to evaluate the effectiveness of key components of our MSDC-Net, including the dilated convolution, MSFCB, and MLFA module.

#### Effectiveness of dilated convolution

First, we trained a network containing only regular convolutions as the baseline. Then, we replace the regular convolutions of the last convolution block with dilated convolutions. Quantitative results are reported in Table 1. As can be seen, the baseline equipped with the ResNet-50 backbone network outperforms that with VGG-16. We attribute this performance gap to the stronger model capacity of ResNet-50. When we replace the regular convolutions of the last convolution block with dilated convolution, we can see further accuracy improvement, where the mIoU increased from 65.2% and 68.3% to 67.5% and 70.8%, respectively. Hence, the dilated convolution contributes considerable improvements over the baseline model by capturing multiscale context information.

**Table 1:** Comparison of networks containing regular convolution and dilated convolution based on different backbones.

Backbone	Methods	Dice (%)	Sen. (%)	Spec. (%)	mIoU (%)
VGG-16	Baseline	70.7	69.9	87.5	65.2
	Dilated convolution	72.6	70.2	87.4	67.5
ResNet-50	Baseline	72.5	71.7	88.5	68.3
	Dilated convolution	74.2	75.1	91.1	70.8

**Figure 8:**
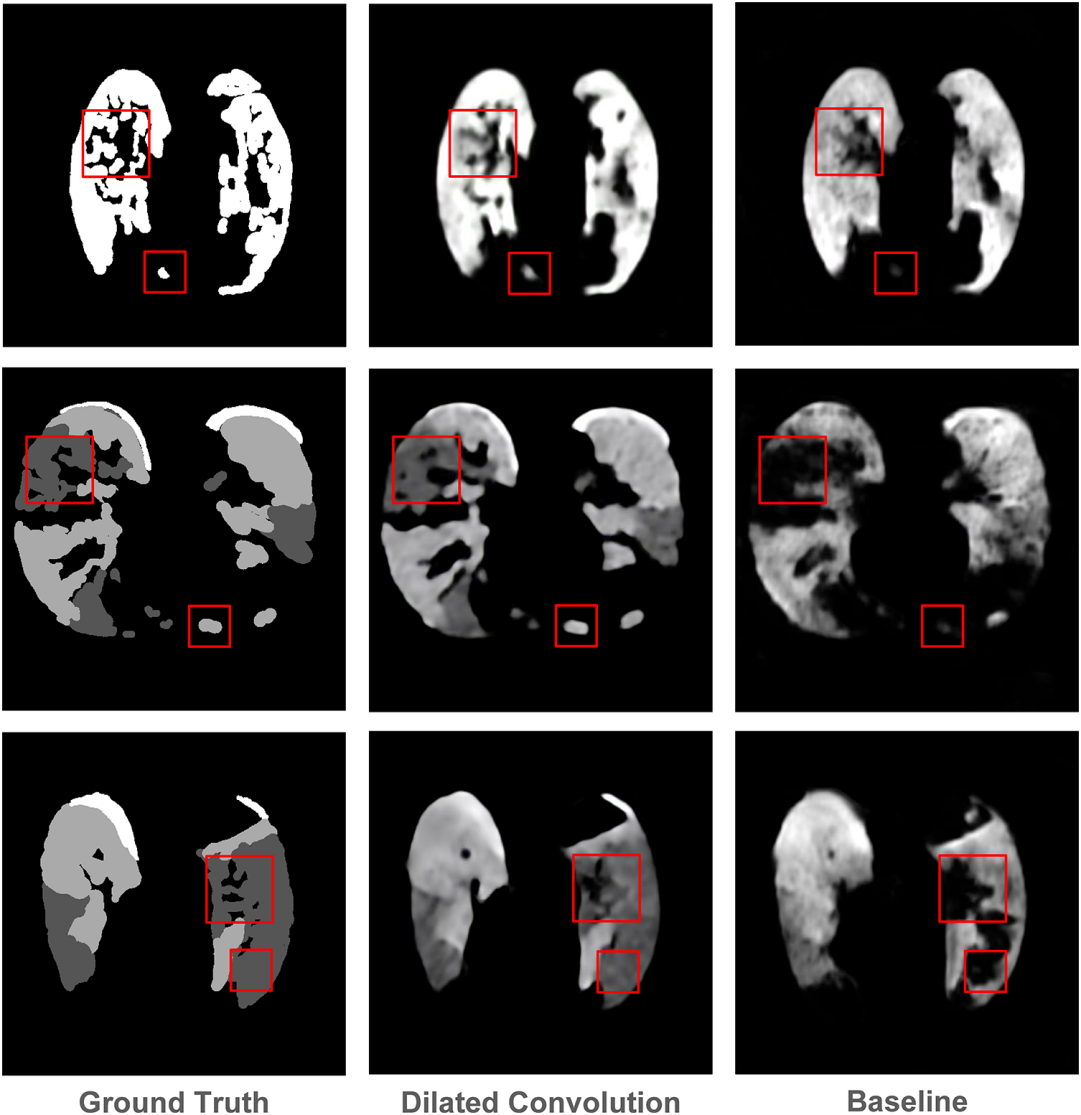
Lung infection segmentation results of different networks, where the ground-glass, consolidation, and pleural effusion are marked in dark gray, bright gray, and white, respectively. The first column is the ground truth. The second column is the segmentation result of the ResNet-50 network using dilated convolution. The last column is the segmentation result of the baseline based on ResNet-50.

In addition, the advantage of the dilated convolution is also confirmed by Fig. 8. We can observe that the network using dilated convolution remarkably outperforms the baseline methods. This demonstrates the ability of dilated convolution to capture and analyze infected lesions of different scales.

#### Number of the dilated convolutions

To further analyze the effectiveness of the dilated convolution, we conduct experiments on how many dilated convolutions can achieve the best segmentation results. In the encoder of our network, there are five blocks, as shown in Fig. 3. In practice, we start with the last block and use the dilated convolution in each block successively. As shown in Table 2, if the dilated convolution is only used in one block, the improvement is marginal (the mIoU score increased from 68.3 to 70.8%). When dilated convolution is used in two blocks, the segmentation performance of the network is obviously improved and achieves the best result in the Dice and mIoU scores. It is worth noting that the use of three dilated convolutions will cause performance degradation, which means that the receptive field of the network is saturated with respect to the input image size. After that, the network performance begins to decline and when dilated convolution is used in the entire encoder, the performance becomes the worst.

**Table 2:** Comparison of networks using a different number of dilated convolutions.

Dilated convolutions used					Dice (%)	Sen. (%)	Spec. (%)	mIoU(%)
MSFCB1	MSFCB2	MSFCB3	MSFCB4	MSFCB5				
					72.5	71.7	88.5	68.3
				✓	74.2	75.1	91.1	70.8
			✓	✓	76.4	76.1	94.7	74.2
		✓	✓	✓	74.4	75.9	90.6	70.1
	✓	✓	✓	✓	68.9	66.5	85.4	64.3
✓	✓	✓	✓	✓	63.8	61.4	82.7	60.2

When excessively dilated convolutions are used in the encoder, the overlarge receptive field of the shallow convolution will weaken its ability to capture local information. In addition, when the receptive field size of the deep convolution is larger than the size of the input image, the filter will degenerate to a 1 × 1 convolution, leading to a decrease in the network performance. In the following experiments, we will use the network in which dilated convolution is utilized within the last two blocks unless stated otherwise.

#### Effectiveness of the MSFCB and MLFA module

To explore the contribution of the proposed MSFCB and MLFA module, we train a network that only uses the original two 3 × 3 convolutions and skip connections as the baseline. Then, we sequentially add the MSFCB, MLFA module, and their combination for joint learning. The experimental results are shown in Table 3. When using MSFCB to replace the original 3 × 3 convolution operation, the mIoU and Dice scores increased from 74.2% and 76.4% to 75.7% and 78.1%, respectively. Moreover, the addition of the MLFA module provides 1.9% and 4.2% improvement of the mIoU and Dice score, while adding both of them can increase mIoU and Dice scores by 4% and 6%, respectively. These improvements demonstrate that our modules are essential to improve performance.

**Table 3:** Comparison of networks with different modules.

Network configuration	Dice (%)	Sen. (%)	Spec. (%)	mIoU (%)
Baseline	76.4	76.1	94.7	74.2
Baseline + MSFCB	78.1	75.6	96.6	75.7
Baseline + MLFA module	80.6	78.5	96.3	76.1
Baseline + MSFCB + MLFA module	82.4	81.1	97.7	78.2

### Comparisons with the state-of-the-arts methods

We compare the proposed MSDC-Net with four state-of-the-art networks of semantic segmentation. Quantitative comparison results of these networks are shown in Table 4. Our MSDC-Net outperforms the compared networks in teams of Dice, sensitivity, and mIoU by a large margin and provides 7.2% and 7.9% higher Dice and mIoU compared to the second-highest score (Dilated-10). It should be noted that the method proposed by^30^ achieves a significant increase in Dice and mIoU values but has a lower specificity score than U-Net^24^ and SegNet^28^, which indicates that the methods proposed by^24,28^ are more conservative. In Table 4, the computational efficiency of different networks is also summarized. It can be noticed that our MSDC-Net has a slightly higher number of parameters compared to other networks but providing a large improvement of performance. In addition, our MSDC-Net also achieved the fastest inference speed among these comparison methods.

**Table 4:** Comparisons with existing methods.

Network	Number of parameters (M)	Inference time (ms)	Dice (%)	Sen. (%)	Spec. (%)	mIoU (%)
FCN^27^	3.8	156	67.9	70.7	85.3	62.5
U-Net^24^	2.4	100	73.3	76.6	98.6	67.1
SegNet^28^	3.1	89.2	70.5	77.8	94.8	65.6
Dilated-10^30^	6.5	78	75.2	78.1	92.3	70.3
MSDC-Net (ours)	5.7	63	82.4	81.1	97.7	78.2

Moreover, quantitative performances on multi-class lesion segmentation, including separate ground-glass, consolidation and pleural effusion region, are summarized in Table 5, where 7.2% improvement in dice score is obtained in ground-glass segmentation, 9.3% improvement in consolidation segmentation and 8.6% improvement in pleural effusion segmentation using our MSDC-Net over the other best-performing methods.

**Table 5:** Comparison of performances on different types of infections (ground-glass, consolidation and pleural effusion).

Network	Ground-glass			Consolidation			Pleural effusion		
	Dice (%)	Sen. (%)	IoU (%)	Dice (%)	Sen. (%)	IoU (%)	Dice (%)	Sen. (%)	IoU (%)
FCN^27^	59.8	65.4	57.4	50.2	60.4	50.0	54.3	65.3	52.6
U-Net^24^	63.5	65.6	60.8	51.1	62.6	50.3	58.3	71.8	53.4
SegNet^28^	62.0	70.5	58.6	55.2	65.5	52.4	60.6	73.3	55.6
Dilated-10^30^	68.6	73.1	66.9	56.2	63.6	53.6	61.6	69.7	59.8
MSDC-Net (ours)	75.8	82.4	71.8	65.5	69.6	62.6	70.2	74.2	67.7

**Figure 9:**
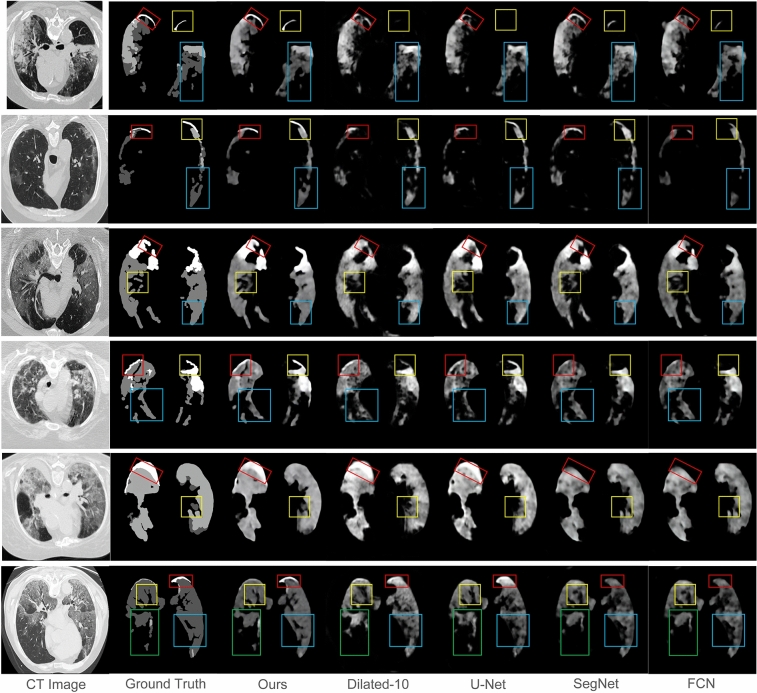
Segmentation results of different scale lesions, where the dark gray, bright gray, and white labels indicate the ground-glass, consolidation, and pleural effusion, respectively.

The segmentation results of our MSDC-Net and other methods, shown in Fig. 9, indicate that our MSDC-Net outperforms other methods remarkably. For example, the first row in Fig. 9 shows the segmentation results of three different sizes of lesions by different methods. It is worth noting that the large, medium, and small-scale lesions marked by three different boxes in Fig. 9 are accurately segmented by our MSDC-Net, which further proves the advantage of our network. In contrast, FCN gives unsatisfactory results, where lesions of various scales cannot be accurately segmented. Dilated-10 and U-Net have improved the segmentation of large lesions and small lesions respectively, but neither of them can accurately segment lesions of various scales at the same time. In addition, the advantage of our MSDC-Net is also confirmed by Fig. 9. As can be seen, our MSDC-Net yields better segmentation result of lesion boundaries than other methods. The success of our MSDC-Net is attributed to the effective use of multiscale and multilevel features, where MSFCBs first capture the multiscale features and then MFA module is employed to aggregate multilevel features for fine segmentation.

## Discussion

In summary, several key components are proposed to enable our network to achieve significant improvements in lesions segmentation of different scales and lesion boundaries. Compared with only recognizing COVID-19 in CT images, lesion segmentation can quantify the number of lesions, lesion volume changes, and lesion density changes, allowing radiologists to quickly understand changes in lesions, and greatly improve the efficiency of radiologists in analyzing changes in the patients' condition. Compared with FCN^27^, the encoder–decoder structure of our method could recover the detailed information during the upsampling process, where the mIoU and Dice scores increased from 62.5% and 67.9% to 78.2% and 82.4%, respectively. Although U-Net^24^ and SegNet^28^ are also based on encoder–decoder structures, MSFCB helps to extract and analyze lesion features of different scales in CT images. Compared with U-Net and SegNet, the mIoU of the proposed model is increased by 11.1% and 12.6%, respectively. Furthermore, unlike Dilated-10^30^, we use a combination of regular convolution and dilated convolution to reduce the computational complexity and add two modules to segment the lesions of different scales more accurately. It is worth noting that compared with other networks, the proposed MSDC-Net has much faster inference speed (0.59 times the average inference time) while providing a great performance improvement (The values of Dice and mIoU increased by 10.6% and 11.8% on average). For example, the proposed MSDC-Net provides the best achievable Dice score (82.4%) and mIoU (78.2%) while consisting of 0.63 × parameters of Dilated-10. The inference time is reduced by 0.093 s compared with FCN. In the case of obtaining the highest performance, the significant increase in inference speed is mainly achieved by frequently adding cross-layer and cross-block connection operations in the network. Therefore, this scheme has a greater advantage over other networks in terms of inference speed.

## Limitations

One of the limitations in this work is that our MSDC-Net focuses on lesion segmentation of COVID-19. Although the accurate segmentation of infected lesions is critical to making treatment decisions, it is often necessary to identify COVID-19 patients before this. Therefore, in the future, we will study a computer-aided diagnosis system that consists of the following three stages: (a) automatic detection of COVID-19 lesions, (b) segmentation of lesions, and (c) quantitative analysis of lesions.

## Conclusion

This paper proposed a MSDC-Net for precise segmentation of infected lesions from CT images. The goal of this paper is to provide an effective and economical tool for faster infection analysis to greatly reduce the spread and massive death toll of COVID-19 through mass-screening and quickly grasp the changes of lesions by quantifying the number, volume and density of lesions. The significant scale difference between different types of COVID-19 lesions and the similarity between the lesions and normal tissues make it different to accurately segment infected lesions. Therefore, we proposed an MSFCB with a series of dilated convolutions to gathering more multiscale context information and introduced an MLFA module for the effective integration of captured multiscale features. Moreover, a MLFA module is used to aggregate features of different levels, which not only effectively replenish context information loss in the repeated downsampling operations but also substantially reduce the semantic gaps between subsequent encoder–decoder. Extensive experiments have been conducted on COVID-19 CT Segmentation dataset analyze the effectiveness of the proposed key modules. The proposed MSDC-Net with several key modules overcomes the limitations of traditional methods that achieved a significant improvement of performance. The results demonstrate that MSFCB can obtain multiscale features of lesions and improve the segmentation accuracy of lesions at different scales, while MLFA module can reduce the information loss in the downsampling process and provide more spatially detailed information when upsampling. Quantitative comparison results showed that our MSDC-Net achieved the best results in the Dice, sensitivity and mIoU and increased the Dice and mIoU values by 10.6% and 11.8%, respectively, on average when compared with the other methods. Qualitative comparison results showed that our MSDC-Net is superior to most existing methods in the segmentation of lesion at various sizes and lesion boundaries. Moreover, it is found that the proposed network is not only effective in COVID lesion segmentation, but also provides a new method and idea for accurately segmenting objects of different sizes at the same time.

## Data Availability

We use a publically dataset of 40 Covid-19 patients, and are available at http://medicalsegmentation.com/covid19/.

## Code availability

We will upload code to Github in the future.
